# Fatal canine parvovirus type 2a and 2c infections in wild Chinese pangolins (*Manis pentadactyla*) in southern China

**DOI:** 10.1111/tbed.14703

**Published:** 2022-09-22

**Authors:** Zhang Lina, Wang Kai, An Fuyu, Zhang Dongliang, Zhang Hailing, Xu Xuelin, Guo Ce, Yan Hongmei, Kuang Yingjie, Zhang Zhidong, Lu Rongguang, Hua Yan

**Affiliations:** ^1^ Eco‐Engineering Department Guangdong Eco‐Engineering Polytechnic Guangzhou China; ^2^ Guangdong Provincial Key Laboratory of Silviculture, Protection and Utilization Guangdong Academy of Forestry Guangzhou China; ^3^ Key Laboratory of Special Animal Epidemic Disease of Ministry of Agriculture and Rural Affairs Institute of Special Animals and Plants Chinese Academy of Agricultural Sciences Changchun China; ^4^ School of Medicine Chinese University of Hongkong Shenzhen Shenzhen China

**Keywords:** canine parvovirus type 2 VP2, Chinese pangolin, isolation and identification, *Manis pentadactyla*

## Abstract

The Chinese pangolin (*Manis pentadactyla*) is a critically endangered scale‐covered mammal belonging to the order Pholidota. Wild pangolins are notably susceptible to pathogen infection and are typically characterized by impoverished health. However, little is currently known regarding the viruses prevalent among pangolins. In this study, we report the detection of two subtypes of canine parvovirus type 2 (CPV‐2), namely CPV‐2a and CPV‐2c, both of which caused severe diarrheal disease in two post‐rescue pangolins with fatal consequences. As in CPV‐2‐infected dogs, intensive lesion of the mucosal layer of the small intestines is a prominent feature in infected pangolins. Moreover, the immunochemistry results demonstrated that CPV‐2 antigens were distributed in the crypts of small intestine. Additionally, typical parvovirus‐associated CPV‐2 were detected after four passages in F81 cells, and typical parvovirus‐like particles, approximately 20 nm in diameter, were observed in the cell supernatants. Phylogenetic analysis revealed that the VP2 viral protein sequences (GenBank accession number OP208805) isolated from one pangolin (termed P1) were classified as CPV‐2c, with 99.8% identity to a CPV‐2c strain (MN832850) isolated from a Taiwanese pangolin found in Taiwan Province. In contrast, VP2 sequences (#OP208806) obtained from the second pangolin (P2) were classified as CPV‐2a, with 99.8% identity to a CPV‐2a strain (KY386858) isolated from southern China. In this study, we thus confirmed the infection of pangolins with CPV‐2c in mainland China and demonstrated that CPV‐2a also can infect pangolins. Based on these findings, we recommend that further investigations should be conducted to establish the interspecies transmission of these viruses among wild pangolins, wild carnivores, and stray dogs.

## 1 INTRODUCTION

The Chinese pangolin (*Manis pentadactyla*) is a scale‐covered mammal belonging to the order Pholidota, which also contains the congeneric Sunda or Malayan pangolin (*Manis javanica*), the Philippine pangolin (*Manis culioensis*), and the Indian or thick‐tailed pangolin (*Manis crassicaudata*) as co‐occurring Asian species (Gaudin et al., 2009). Pangolins have been established to play pivotal ecological roles in the structure and function of forests in southern China, and have accordingly been identified as key indicator species, the status of which closely reflects that of ecosystem health. However, as a consequence of widespread poaching and trafficking, the number of pangolins has decreased substantially in recent years, and across much of their distribution range, these mammals are believed to be critically endangered (Wang et al., 2019).

The continued survival of wild pangolin populations is further jeopardized by their generally poor health state, which is believed to be attributable to pathogen infections. In this regard, it has been reported that common etiological agents, such as canine parvovirus (CPV‐2), canine distemper virus (CDV), and parainfluenza virus 5 (PIV5), which are highly prevalent among mammals in the families Felidae and Canidae, can infect Pangolins (Chin et al., 2015; Wang et al., 2020, 2019). Moreover, viral metagenomic analyses have revealed the presence of coronavirus and Sendai virus sequences in pangolins (Liu et al., 2019).

CPV‐2, a member of the family *Parvoviridae*, genus *Protoparvovirus*, and species *Carnivore protoparvoviru*s *1* (Cotmore et al., 2014), has been characterized as an autonomously replicating negative‐sense single‐stranded non‐enveloped DNA virus, with an icosahedral capsid protein of approximately 20 nm in diameter (Organtini et al., 2015). The viral genome is approximately 5200 bases in length and consists of two open reading frames (ORFs) with palindromic structures at the 3ʹ and 5ʹ ends. ORF2 encodes the VP1 and VP2 viral capsid proteins, the latter of which, consisting of 584 amino acids, is the major capsid proteins that determines viral host range, antigenicity, and hemagglutination properties (Sehata et al., 2017). With respect to infectivity, it has been established that the residue at position 300 of VP2 contributes to determining the host range (Allison et al., 2016).

Given its rapid rate of nucleotide replacement, CPV‐2 is continually evolving as a globally spreading virus (Shackelton et al., 2005), and at present, four major CPV‐2 subtypes, namely CPV‐2, CPV‐2a, CPV‐2b, and CPV‐2c, are recognized worldwide (Behdenna et al., 2019; Mira et al., 2019; Geng et al., 2015; Decaro et al., 2009; Li et al., 2019; De la Torre et al., 2018). Although transmissions of subtype CPV‐2 and its variants among wild carnivores, particularly those in the families *Felidae* and *Canidae*, are commonplace (Hoelzer & Parrish, 2010; Voorhees et al., 2019), CPV‐2 infections in animals belonging to the order Pholidota are rarely reported. Recently, however, Wang et al. (2020) have reported that CPV‐2c subtype parvovirus can cause severe diarrhoea‐associated diseases in Taiwanese pangolins, and in this study, we describe the discovery of fatal CPV‐2a and CPV‐2c infection in two Chinese pangolins rescued from forests in southern China.

## 2 MATERIALS AND METHODS

### 2.1 Sample collection

Two sub‐adult female Chinese pangolins (bodyweight: 1.1 kg), designated P1 and P2, were found by local inhabitants in areas of natural forest and taken to the Guangdong Wildlife Rescue Center (Guangzhou), although both subsequently died within a week of rescue.

### 2.2 Pathological observation and immunohistochemistry

Following post‐mortem examination conducted at the Guangdong Wildlife Rescue Center, entailing gross observations, tissues were collected from different parts of the intestines, including the duodenum, jejunum, ileum, colon, rectum, and mesenteric lymph nodes, as well from other major organs. These tissues were fixed in 10% neutral formalin, then sliced to give 5‐μm sections, which were subsequently processed for haematoxylin and eosin staining or immunochemical analyses using standard protocols. Immunochemistry was performed using in‐house CPV‐2 monoclonal antibody (mouse‐origin; twofold dilution) as the primary antibody and horseradish peroxide (HRP)‐conjugated anti‐goat IgG (Sigma; 1:1000 dilution) as the secondary antibody. The histological features and immunohistochemical results were observed and images were captured using an Olympus light microscope.

### 2.3 Virus screening, isolation, and transmission electron microscopy observations

Viruses known to be associated with diarrheal diseases in pangolins, including CPV‐2, canine coronavirus (CCoV), canine distemper virus (CDV), and dog circovirus (DogCV), were screened based on PCR analyses using previously described primers (Deng et al., 2018; Hsu et al., 2016). For the purposes of virus isolation, samples of pangolin tissues were homogenized, diluted with phosphate‐buffered saline, and purified by passing through a 0.22‐μm sterile filter. These filtered samples were subsequently added to monolayers of F81 cells (ATCC‐CL‐0081) maintained in minimum essential media supplemented with 10% foetal bovine serum at an amount equal to 10% of the cell culture medium volume. The cells were thereafter inspected daily for cytopathic effects. Cells showing evidence of infection were collected, purified by ultracentrifugation, and following negative staining with phosphotungstic acid, subjected to transmission electron microscopy (Hitachi).

### 2.4 ORF 2 sequence amplification and phylogeny

Sequences of the VP2 protein were PCR amplified using previously described primers (Lu et al., 2020), and sequences of the amplicons thus obtained were verified using the Sanger method (Sangon). For phylogenetic analysis, we constructed a tree based on the maximum‐likelihood (ML) method inferred using 144 *Carnivore protoparvovirus 1* VP2 gene sequences (Table S1). These sequences were aligned using Clustal W (Larkin et al., 2007), and the ML tree was generated using MEGA X software (Kumar et al., 2016) based on 3‐parameter (T92) model and gamma distribution with invariant sites (G + I) (Tamura & Nei, 1993) as the most fittest condition. The reliability of the ML tree was assessed based on 500 bootstrap replicates, and the original tree was summarized and annotated using Figtree software.

## 3 RESULTS AND DISCUSSION

### 3.1 Clinical symptoms and pathological lesions

**FIGURE 1:**
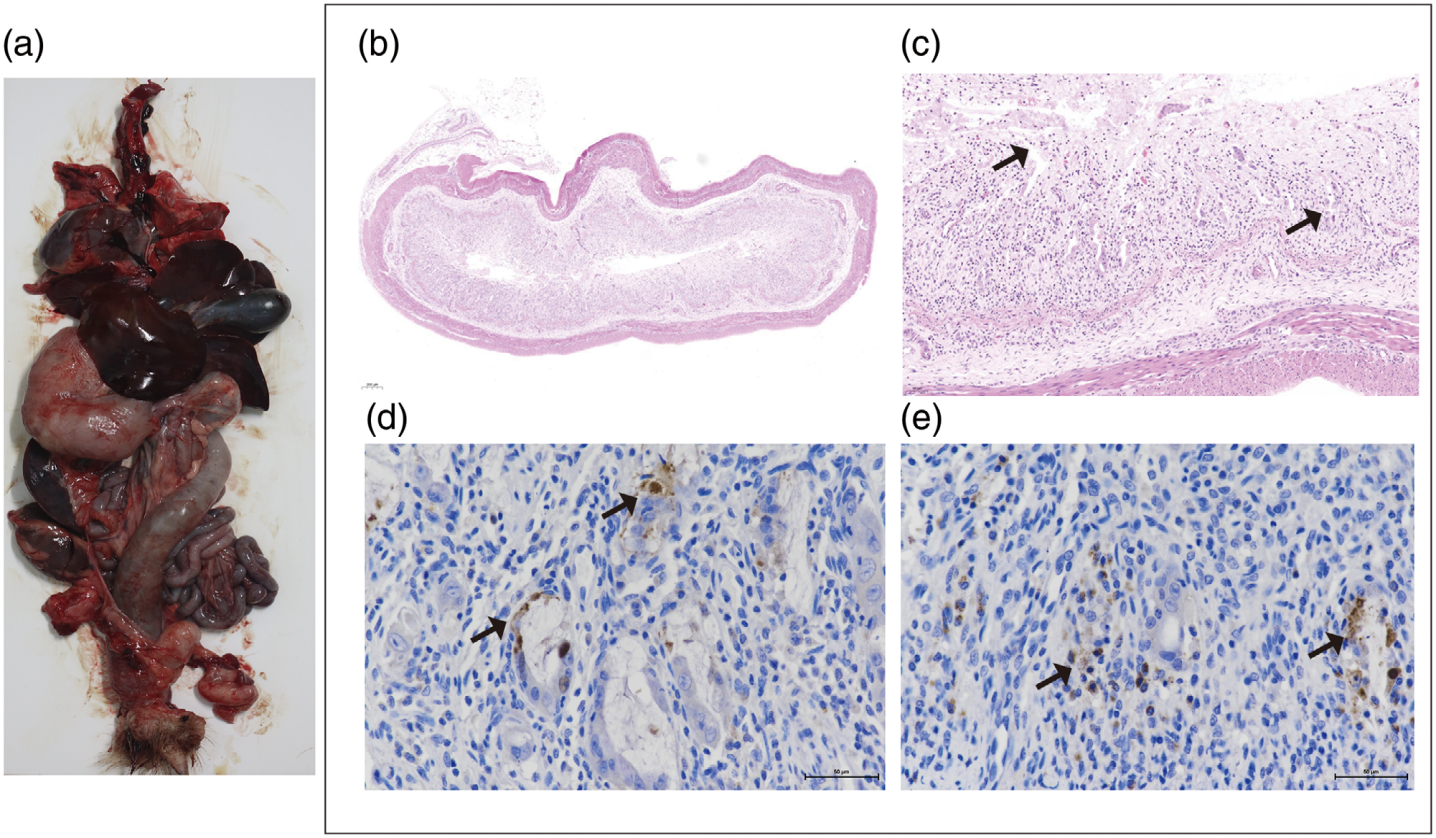
Pathological lesions and immunohistochemical analyses of CPV‐2‐infected pangolins (sub‐adult females) suffering from severe diarrhoea. Representative images of gross changes and histopathological lesions are shown. Panel (a) presents a gross observation of the internal organs, showing apparent congestion and haemorrhage. Panels (b) and (c) show histopathological lesions in the jejunum. Pronounced injury in the mucosa layer of small intestine is a prominent feature, notably the necrosis and shedding of the intestinal mucosal intraepithelial cells and glands. Panels (d) and (e) show the immunohistochemistry results for the jejunum and ileum, respectively. The black arrows indicate the significant pathological lesion or CPV‐2 antigen‐positive area. Scale bars are shown in the lower right‐hand corners.

General lethargy, reduced food intake, and loose stools were among the symptoms observed in the two pangolins examined in the present study. Furthermore, although electrolyte disturbance was observed, there was no evidence of leukopenia. The pangolins died naturally at 4–6 days post‐rescue, with post‐mortem examinations revealing apparent congestion and haemorrhage in the intestinal tract, thickened intestinal walls, and necrosis of the mucosal tissue (Figure 1a). Histopathological observations, performed to determine lesions associated with CPV‐2‐induced diarrhoea, revealed intensive injury to the mucosal layer of the small intestine to be a prominent feature, particularly the necrosis and shedding of intestinal mucosal intraepithelial cells and glands, which were observed in all parts of the small intestine (Figure 1b,c). Moreover, the submucosal layers were found to be characterized by inflammatory infiltrates, comprising primarily neutrophils and scattered lymphocytes. In addition, we observed a reduction in the number of lymphocytes in the spleen. The detection of bacteria in rectal tissues provided evidence to indicate the probable occurrence of secondary bacterial infection. In contrast, there was no indication of histopathological changes in the heart, liver, lungs, or kidneys.

Immunohistochemical analyses were performed to determine the localization of CPV‐2 antigen in the intestinal system of the CPV‐2‐infected pangolins, representative high‐magnification images of which are presented in Figure 1d,e. CPV‐2 antigens were detected in the duodenum, jejunum, and ileum, although not in the mesenteric lymph nodes. Strong HRP signals were observed in the crypt region and vicinity of inflamed cells, indicating that CPV‐2 antigens were distributed in these areas. These pathological changes in CPV‐2‐infected pangolins are similar to those previously described in CPV‐2‐infected dogs, in which haemorrhagic enteritis and crypt neurosis are prominent features (Pollock, 1982).

Moreover, consistent with our observations of histopathological lesions, the crypt was established to be the major CPV‐2 antigen‐positive region (Figure 1), indicating that CPV‐2 targets and replicates within the intestinal tract cells of animals in the order Pholidota.

### 3.2 Virus screening, isolation, and transmission electron microscopy observations

**FIGURE 2:**
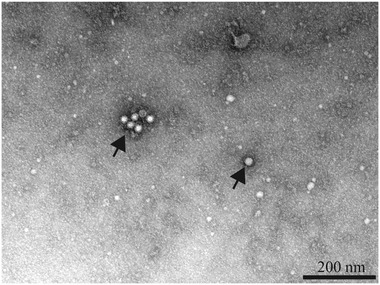
A transmission electron micrograph showing a group of parvo‐like virus particles, indicated by the back arrows

The findings of our PCR analyses revealed that samples obtained from the two pangolins were CPV‐2‐positive, whereas there was no evidence to indicate infection with CCoV, CDV, or DogCV. Moreover, typical parvovirus‐associated CPV‐2 were detected after four passages in F81 cells, and typical parvovirus‐like particles, approximately 20 nm in diameter, were observed in the cell supernatants (Figure 2). Based on these observations, we believe CPV‐2s to be the etiological agents causing pangolin diarrhoea.

### 3.3 Phylogeny and VP2 amino acid sequence substitutions

**FIGURE 3:**
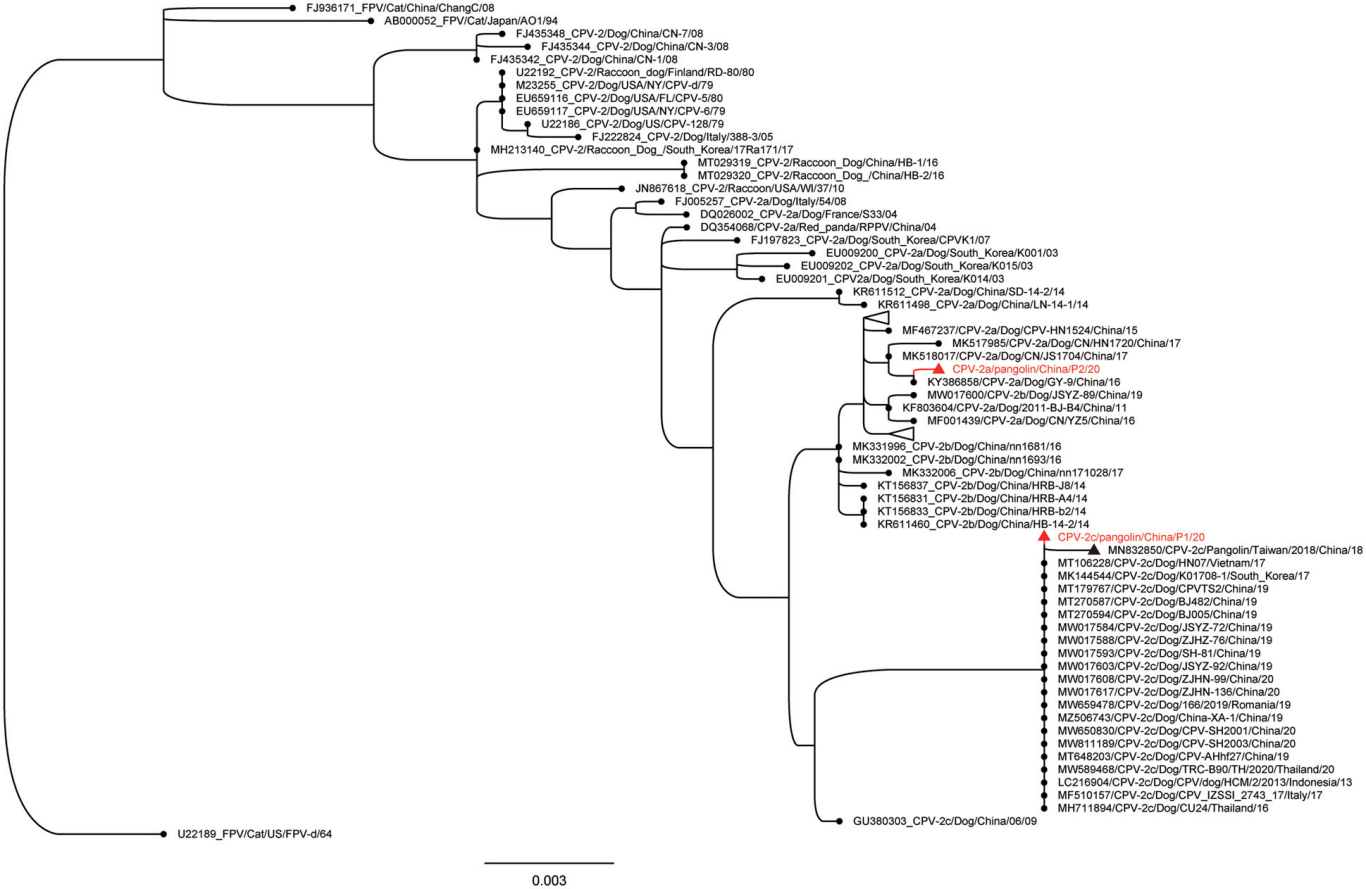
Phylogenetic analysis of VP2 gene sequences from pangolin and related parvoviruses. The ML tree was constructed based on VP2 gene sequences of 142 parvoviruses belonging to the species *Carnivore protoparvovirus 1* (FPV [*n* = 3]; CPV‐2 [*n* = 14] or its variants CPV‐2a [*n* = 68], CPV‐2b [*n* = 8], or CPV‐2c [*n* = 52]). The VP2 gene sequences of FPV were used as an outgroup and rooted by the oldest FPV sequence (FPV/Cat/US/FPV‐d/64, U22189). The two pangolin parvovirus isolates obtained in this study are indicated by red triangle, and the reference virus is denoted by a black triangle. Details of the several subclades grouped close to CPV‐2b or CPV‐2c are presented in Figure S1. The scale bar is shown at the bottom of the tree denotes.

The original tree was shown in Figure S1, and a summarized tree is shown in Figure 3. The VP2 sequences in samples obtained from pangolin P1 were classified as CPV‐2c subclade, with 99.8% identity to a CPV‐2c strain (MN832850) isolated from a Taiwanese pangolin in China, whereas those from pangolin P2 were classified as CPV‐2a subclade, with 99.8% identity to a CPV‐2a strain (KY386858). Notably, CPV‐2a and CPV‐2c are the dominant strains prevalent among domestic dogs in southern China (Chiang et al., 2016; Hao et al., 2020; Qi et al., 2020) and in neighbouring tropical countries, respectively (Charoenkul et al., 2019; Hoang et al., 2019; Inthong et al., 2020). In the present study, we established that the two Chinese pangolins were infected with CPV‐2a and CPV‐2c, respectively. The VP2 sequences obtained from pangolin P2 were classified into the CPV‐2a subtype group, closely related to CPV‐2a strains, such as KY386858, MK518017, and MK517985 isolated from dogs in Guiyang, Jiangsu, and Henan provinces in the central and southern regions of China (Figure 3). Moreover, the VP2 sequences obtained from pangolin P1 were classified into the CPV‐2c subtype group, closely related to CPV‐2c strains, which were isolated in the neighbouring countries of Vietnam and South Korea (Figure 3).

The amino acid sequence of the VP2 viral protein detected in pangolin P2 is characterized by an asparagine (N) residue at position 426 and refers to other residues (such as residues 80, 103, and 297) in the VP2 protein, same as CPV‐2a strains. In contrast, the sequence in pangolin P1 has a glutamic acid (E) residue at position 426, same as CPV‐2c strains. Consistently, the P2 sequence has glutamine (Q) and alanine (A) residues at positions 370 and 440, respectively, whereas the P1 sequence is characterized by arginine (R) and threonine (T) residues at positions 370 and 440.

Most CPV‐2 variants are characterized by a glycine residue at position 300 of the VP2 sequence, which has been demonstrated to be necessary for the infection of canine species (Allison et al., 2016; Voorhees et al., 2019). The findings of numerous studies have indicated that the CPV‐2a variant can infect wild civet cats (*Paradoxurus musangus*) (Mendenhall et al., 2016), red pandas (*Ailurus fulgens*) (Qin et al., 2007), masked palm civets (*Paguma larvata*), and Chinese ferret badgers (*Melogale moschata*) (Chang & Chen, 2021) in East Asian countries. The CPV‐2c variant has also been confirmed to infect masked palm civets and Chinese ferret badgers (Chang & Chen, 2021). Moreover, CPV‐2 has been established to be highly prevalent in farmed raccoon dogs (Lu et al., 2020).

Pangolins remain critically endangered worldwide, owing to a range of threats, not least of which is pathogen infection. To date, however, there have been a few studies that have examined the pathogens infecting pangolins. In this study, however, we were able to confirm CPV‐2c infection among pangolins inhabiting mainland China and established that these animals can also be infected with the CPV‐2a variant. In this regard, the findings of previous studies have provided evidence to indicate that CPV‐2 frequently undergoes interspecies transmission among domestic and wild animals, with asymptomatic individuals (e.g. stray dogs and raccoons) inhabiting the peripheries of urban areas acting as virus reservoirs that promote the spread of CPV‐2 to wild animals (Allison et al., 2012, 2013; Hirsch et al., 2013). Notably, the two strains detected in this study are phylogenetically close to CPV‐2 strains derived from dogs. Moreover, stray dogs have been observed attacking wild pangolins in areas in which urban development has encroached on the habitats of these animals (Wang et al., 2020), thereby indicating the potential for cross‐species transmission between stray dogs and wild pangolins.

In summary, in this study, we report the infection of Chinese pangolins with two strains of canine parvovirus type 2 causing diarrheal diseases. These two CPV‐2 strains are closely related to CPV‐2a and CPV‐2c, which are prevalent in China and neighbouring countries, respectively, thereby revealing that the CPV‐2 strains infecting Chinese pangolins are close to those known to be of dog origin. Based on our findings, we recommend that further investigations should be conducted to assess the potential interspecies transmission among wild Chinese pangolins and domestic or feral dogs.

## AUTHOR CONTRIBUTIONS

Lu Rongguang and Hua Yan conceived the study and designed the experiments. Zhang Lina and Wang Kai wrote the manuscript. An Fuyu, Zhang Dongliang, Zhang Hailing, Xu Xuelin, Guo Ce, Yan Hongmei, Kuang Yingjie, and Zhang Zhidong carried out the animal experiment, sample collection, and sample analysis. All authors read and approved the final manuscript.

## CONFLICT OF INTEREST

The authors declare no conflict of interest.

## ETHICS STATEMENT

The Pangolins were treated using the highest standards of care at the rescue centre in all steps of diagnosis and treatment. Tissue samples were collected only from dead rescued pangolins.

## Supporting information

Fig S1. Phylogenetic analysis of VP2 gene sequences from pangolin and related parvoviruses.
Table. S1 Data of reference sequences (VP2 gene)

## Data Availability

The data that support the findings of this study are openly available in the GenBank database at https://www.ncbi.nlm.nih.gov/nucleotide/ under accession numbers.
