# Supplementary material for: Fatal canine parvovirus type 2a and 2c infections in wild Chinese pangolins (Manis pentadactyla) in southern China
Source: Transbound Emerg Dis. 2022 Sep 22;69(6):4002–8. doi: 10.1111/tbed.14703 (PMC10087772; doi:10.1111/tbed.14703)
Supplement: Supplementary file 1 — Fig S1. Phylogenetic analysis of VP2 gene sequences from pangolin and related parvoviruses. Table. S1 Data of reference sequences (VP2 gene) [file TBED-69-4002-s001.docx]

**Fatal canine parvovirus type 2a and 2c infections in wild Chinese pangolins (*Manis pentadactyla*) in** **southern China**

Zhang Lina ^1^, Wang Kai^2^, An Fuyu^2^, Zhang Dongliang^3^, Zhang Hailing^3^, Xu Xuelin^2^, Guo Ce^2^, Yan Hongmei^2^, Kuang Yingjie^2^, Zhang Zhidong^2^, Lu Rongguang^4^*, Hua Yan^2^*****

1. Eco‐Engineering Department, Guangdong Eco‐Engineering Polytechnic, Guangzhou 510520, China

2. Guangdong Provincial Key Laboratory of Silviculture, Protection and Utilization, Guangdong Academy of Forestry, Guangzhou 510520, China

3. Key Laboratory of Special Animal Epidemic Disease of Ministry of Agriculture and Rural Affairs, Institute of Special Animals and Plants, Chinese Academy of Agricultural Sciences, No. 4899 Juye Street Changchun 130112, Jilin Province, China

4. School of Medicine, Chinese University of Hongkong Shenzhen, No.2001 Longxiang Avenue, Shenzhen 518172, Guangdong Province, China

*Correspondence1: [wildlife530@hotmail.com](mailto:wildlife530@hotmail.com)

Eco‐Engineering Department, Guangdong Eco‐Engineering Polytechnic, Guangzhou 510520, China.

*Correspondence2: lurongguang@cuhk.edu.cn

School of Life and Health Sciences, Chinese University of Hongkong Shenzhen, No.2001 Longxiang Avenue, Shenzhen 518172, Guangdong Province, China.


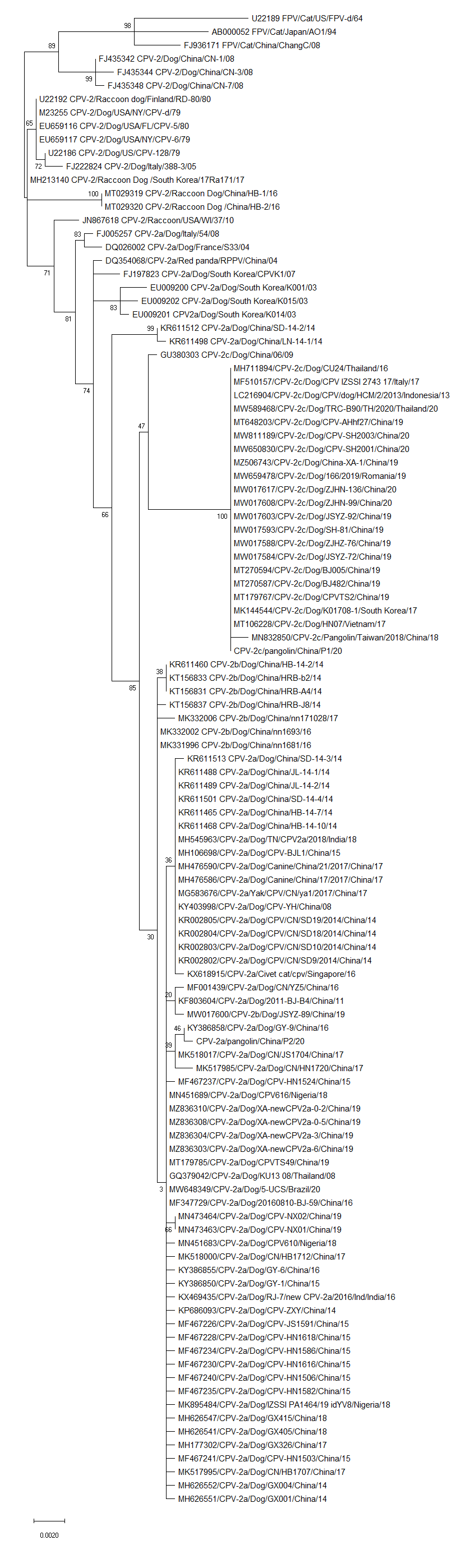


**Fig S1.** Phylogenetic analysis of VP2 gene sequences from pangolin and related parvoviruses. The ML tree was constructed based on VP2 gene sequences of 142 parvoviruses belonging to the species *Carnivore protoparvovirus 1* (FPV [n = 3]; CPV-2 [n = 14] or its variants CPV-2a [n = 68], CPV-2b [n = 8], or CPV-2c [n = 52]). The VP2 gene sequences of FPV were used as an outgroup and rooted by the oldest FPV sequence (FPV/Cat/US/FPV-d/64, U22189).

**Table. S1 Data of reference sequences (VP2 gene)**

|  |  | Accession no. | Isolate name |
| --- | --- | --- | --- |
| **FPV group** |  | U22189 | FPV/Cat/US/FPV-d/64 |
|  |  | AB000052 | FPV/Cat/Japan/AO1/94 |
|  |  | FJ936171 | FPV/Cat/China/ChangC/08 |
|  |  |  |  |
| **CPV-2 group** | 1 | U22192 | CPV-2/Raccoon_dog/Finland/RD-80/80 |
|  | 2 | M23255 | CPV-2/Dog/USA/NY/CPV-d/79 |
|  | 3 | EU659116 | CPV-2/Dog/USA/FL/CPV-5/80 |
|  | 4 | EU659117 | CPV-2/Dog/USA/NY/CPV-6/79 |
|  | 5 | U22186 | CPV-2/Dog/US/CPV-128/79 |
|  | 6 | FJ222824 | CPV-2/Dog/Italy/388-3/05 |
|  | 7 | FJ435342 | CPV-2/Dog/China/CN-1/08 |
|  | 8 | FJ435344 | CPV-2/Dog/China/CN-3/08 |
|  | 9 | FJ435348 | CPV-2/Dog/China/CN-7/08 |
|  | 10 | JN867618 | CPV-2/Raccoon/USA/WI/37/10 |
|  | 11 | MH213135 | CPV-2/Raccoon_Dog /South_Korea/17Ra5/17 |
|  | 12 | MH213140 | CPV-2/Raccoon_Dog /South_Korea/17Ra171/17 |
|  | 13 | MT029319 | CPV-2/Raccoon_Dog /China/HB-1/16 |
|  | 14 | MT029320 | CPV-2/Raccoon_Dog /China/HB-2/16 |
|  |  |  |  |
| **CPV-2a group** |  |  |  |
|  | 1 | M24000 | CPV-2a/Dog/USA/VA/CPV-31/83 |
|  | 2 | FJ869128 | CPV-2a/Dog/Thailand/KU11/04 |
|  | 3 | FJ005257 | CPV-2a/Dog/Italy/54/08 |
|  | 4 | DQ026002 | CPV-2a/Dog/France/S33/04 |
|  | 5 | AB054215 | CPV-2a/Cat/Vietnam/V120/97 |
|  | 6 | EU009200 | CPV-2a/Dog/South_Korea/K001/03 |
|  | 7 | EU009201 | CPV-2a/Dog/South_Korea/K014/03 |
|  | 8 | EU009202 | CPV-2a/Dog/South_Korea/K015/03 |
|  | 9 | FJ197823 | CPV-2a/Dog/South_Korea/CPVK1/07 |
|  | 10 | KR611512 | CPV-2a/Dog/China/ SD-14-2/14 |
|  | 11 | KR611513 | CPV-2a/Dog/China/ SD-14-3/14 |
|  | 12 | KR611488 | CPV-2a/Dog/China/ JL-14-1/14 |
|  | 13 | KR611489 | CPV-2a/Dog/China/ JL-14-2/14 |
|  | 14 | KR611498 | CPV-2a/Dog/China/ LN-14-1/14 |
|  | 15 | KR611501 | CPV-2a/Dog/China/ SD-14-4/14 |
|  | 16 | KR611465 | CPV-2a/Dog/China/HB-14-7/14 |
|  | 17 | KR611468 | CPV-2a/Dog/China/HB-14-10/14 |
|  | 18  19  20  21  22  23  24  25  26  27  28  29  30  31  32  33  34  35  36  37  38  39  40  41  42  43  44  45  46  47  48  49  50  51  52  53  54  55  56  57  58  59  60  61  62  63  64  65  66  67  68 | \| KY386858 \| \| --- \| \| MK518017 \| \| MN451689 \| \| MF001439 \| \| MZ836310 \| \| MZ836308 \| \| MZ836304 \| \| MZ836303 \| \| KF803604 \| \| MW017600 \| \| MT179785 \| \| GQ379042 \| \| MW648349 \| \| MN473464 \| \| MN473463 \| \| MN451683 \| \| MK895484 \| \| MK518000 \| \| MK517995 \| \| MK517985 \| \| MH545963 \| \| MH106698 \| \| MH476590 \| \| MH476586 \| \| MH626552 \| \| MH626551 \| \| MH626547 \| \| MH626541 \| \| MH177302 \| \| MF347729 \| \| MG583676 \| \| KY403998 \| \| MF467241 \| \| MF467240 \| \| MF467237 \| \| MF467235 \| \| MF467234 \| \| MF467230 \| \| MF467228 \| \| MF467226 \| \| KY386855 \| \| KY386850 \| \| KX469435 \| \| KR002805 \| \| KR002804 \| \| KR002803 \| \| KR002802 \| \| KP686093 \| \| DQ354068 \| \| KX618915 \|   M24003 | \| CPV-2a/Dog/China/GY-9/16 \| \| --- \| \| CPV-2a/Dog/China/CN/JS1704/17 \| \| CPV-2a/Dog/Nigeria/CPV616/18 \| \| CPV-2a/Dog/China/CN/YZ5/16 \| \| CPV-2a/Dog/China/XA-newCPV2a-0-2/19 \| \| CPV-2a/Dog/China/XA-newCPV2a-0-5/19 \| \| CPV-2a/Dog/China/XA-newCPV2a-3/19 \| \| CPV-2a/Dog/China/XA-newCPV2a-6/19 \| \| CPV-2a/Dog/China/2011-BJ-B4/11 \| \| CPV-2b/Dog/China/JSYZ-89/19 \| \| CPV-2a/Dog/China/CPVTS49/19 \| \| CPV-2a/Dog/Thailand/KU13_08/08 \| \| CPV-2a/Dog/Brazil/5-UCS/20 \| \| CPV-2a/Dog/China/CPV-NX02/19 \| \| CPV-2a/Dog/China/CPV-NX01/19 \| \| CPV-2a/Dog/Nigeria/CPV610/18 \| \| CPV-2a/Dog/Nigeria/IZSSI_PA1464/19_idYV8/18 \| \| CPV-2a/Dog/China/CN/HB1712/17 \| \| CPV-2a/Dog/China/CN/HB1707/17 \| \| CPV-2a/Dog/China/CN/HN1720/17 \| \| CPV-2a/Dog/India/TN/CPV2a/2018/18 \| \| CPV-2a/Dog/China/CPV-BJL1/15 \| \| CPV-2a/Dog/China/Canine/China/21/2017/17 \| \| CPV-2a/Dog/China/Canine/China/17/2017/17 \| \| CPV-2a/Dog/China/GX004/14 \| \| CPV-2a/Dog/China/GX001/14 \| \| CPV-2a/Dog/China/GX415/18 \| \| CPV-2a/Dog/China/GX405/18 \| \| CPV-2a/Dog/China/GX326/17 \| \| CPV-2a/Dog/China/20160810-BJ-59/16 \| \| CPV-2a/Yak/China/CPV/CN/ya1/2017/17 \| \| CPV-2a/Dog/China/CPV-YH/08 \| \| CPV-2a/Dog/China/CPV-HN1503/15 \| \| CPV-2a/Dog/China/CPV-HN1506/15 \| \| CPV-2a/Dog/China/CPV-HN1524/15 \| \| CPV-2a/Dog/China/CPV-HN1582/15 \| \| CPV-2a/Dog/China/CPV-HN1586/15 \| \| CPV-2a/Dog/China/CPV-HN1616/15 \| \| CPV-2a/Dog/China/CPV-HN1618/15 \| \| CPV-2a/Dog/China/CPV-JS1591/15 \| \| CPV-2a/Dog/China/GY-6/16 \| \| CPV-2a/Dog/China/GY-1/15 \| \| CPV-2a/Dog/India/RJ-7/new CPV-2a/2016/Ind/16 \| \| CPV-2a/Dog/China/CPV/CN/SD19/2014/14 \| \| CPV-2a/Dog/China/CPV/CN/SD18/2014/14 \| \| CPV-2a/Dog/China/CPV/CN/SD10/2014/14 \| \| CPV-2a/Dog/China/CPV/CN/SD9/2014/14 \| \| CPV-2a/Dog/China/CPV-ZXY/14 \| \| CPV-2a/Red panda/China/RPPV/04 \| \| CPV-2a/Civet cat/Singapore/CPV/16 \|   CPV-2a/Dog/USA/IL/CPV-15/84 |
| **CPV-2b group** |  |  |  |
|  | 1 | KR611459 | CPV-2b/Dog/China/HB-14-1/14 |
|  | 2 | KR611460 | CPV-2b/Dog/China/HB-14-2/14 |
|  | 3  4  5  6  7  8 | KT156837  KT156833  MK332006  MK332002  MK331996  MH660524 | \| CPV-2b/Dog/China/HRB-J8/14 \| \| --- \| \| CPV-2b/Dog/China/HRB-b2/14 \| \| CPV-2b/Dog/China/nn171028/17 \| \| CPV-2b/Dog/China/nn1693/16 \| \| CPV-2b/Dog/China/nn1681/16 \| \| CPV-2b/Dog/China/N2/18 \| |
|  |  |  |  |
|  |  |  |  |
| **CPV-2c group** |  |  |  |
|  | 1 | FJ005248 | CPV-2c/Dog/Italy/219-2/08 |
|  | 2 | DQ025988 | CPV-2c/Dog/France/S19/04 |
|  | 3 | FJ005199 | CPV-2c/Dog/Germany/G172/97 |
|  | 4 | FJ005246 | CPV-2c/Dog/Spain/128/08 |
|  | 5 | GU380303 | CPV-2c/Dog/China/06/09 |
|  | 6 | GU380305 | CPV-2c/Dog/China/08/09 |
|  | 7  8  9  10  11  12  13  14  15  16  17  18  19  20  21  22  23  24  25  26  27  28  29  30  31  32  33  34  35  36  37  38  39  40  41  42  43  44  45  46  47  48  49  50  51  52 | \| MN451682 \| \| --- \| \| MN451678 \| \| MG013488 \| \| MH711902 \| \| MH711894 \| \| MH476587 \| \| MH476583 \| \| MF510157 \| \| LC216904 \| \| MW589468 \| \| MT840294 \| \| MT840293 \| \| MT648203 \| \| MW811189 \| \| MW650830 \| \| MZ506743 \| \| MW659478 \| \| MW659477 \| \| MW659476 \| \| MW659475 \| \| MW659474 \| \| MW659473 \| \| MW659472 \| \| MW659471 \| \| MW659470 \| \| MW659469 \| \| MW017617 \| \| MW017608 \| \| MW017603 \| \| MW017597 \| \| MW017593 \| \| MW017592 \| \| MW017591 \| \| MW017590 \| \| MW017588 \| \| MW017587 \| \| MW017586 \| \| MW017585 \| \| MW017584 \| \| MW017581 \| \| MT270594 \| \| MT270587 \| \| MT179767 \| \| MK144544 \| \| MT106228 \| \| MN832850 \| | \| CPV-2c/Dog/Nigeria/CPV609/18 \| \| --- \| \| CPV-2c/Dog/Nigeria/CPV605/18 \| \| CPV-2c/Dog/China/CPV-SH1516/17 \| \| CPV-2c/Cat/Thailand/CU21/16 \| \| CPV-2c/Dog/Thailand/CU24/16 \| \| CPV-2c/Dog/China/Canine/China/18/2017/17 \| \| CPV-2c/Dog/China/Canine/China/14/2017/17 \| \| CPV-2c/Dog/Italy/CPV_IZSSI_2743_17/17 \| \| CPV-2c/Dog/Indonesia/CPV/dog/HCM/2/2013/13 \| \| CPV-2c/Dog/Thailand/TRC-B90/TH/2020/20 \| \| CPV-2c/Dog/Nigeria/IZSSI_PA1464/19_idYV7_TR_4A72/18 \| \| CPV-2c/Dog/Nigeria/IZSSI_PA1464/19_idV4_TR_4A72/18 \| \| CPV-2c/Dog/China/CPV-AHhf27/19 \| \| CPV-2c/Dog/China/CPV-SH2003/20 \| \| CPV-2c/Dog/China/CPV-SH2001/20 \| \| CPV-2c/Dog/China/China-XA-1/19 \| \| CPV-2c/Dog/Romania/166/2019/19 \| \| CPV-2c/Dog/Romania/165/2019/19 \| \| CPV-2c/Dog/Romania/164/2019/19 \| \| CPV-2c/Dog/Romania/163/2019/19 \| \| CPV-2c/Dog/Romania/162/2019/19 \| \| CPV-2c/Dog/Romania/161/2019/19 \| \| CPV-2c/Dog/Romania/160/2019/19 \| \| CPV-2c/Dog/Romania/159/2019/19 \| \| CPV-2c/Dog/Romania/158/2019/19 \| \| CPV-2c/Dog/Romania/157/2019/19 \| \| CPV-2c/Dog/China/ZJHN-136/20 \| \| CPV-2c/Dog/China/ZJHN-99/20 \| \| CPV-2c/Dog/China/JSYZ-92/19 \| \| CPV-2c/Dog/China/JSYZ-86/19 \| \| CPV-2c/Dog/China/SH-81/19 \| \| CPV-2c/Dog/China/SH-80/19 \| \| CPV-2c/Dog/China/SH-79/19 \| \| CPV-2c/Dog/China/SH-78/19 \| \| CPV-2c/Dog/China/ZJHZ-76/19 \| \| CPV-2c/Dog/China/ZJHZ-75/19 \| \| CPV-2c/Dog/China/ZJHZ-74/19 \| \| CPV-2c/Dog/China/ZJHZ-73/19 \| \| CPV-2c/Dog/China/JSYZ-72/19 \| \| CPV-2c/Dog/China/JSYZ-69/19 \| \| CPV-2c/Dog/China/BJ005/19 \| \| CPV-2c/Dog/China/BJ482/19 \| \| CPV-2c/Dog/China/CPVTS2/19 \| \| CPV-2c/Dog/South Korea/K01708-1/17 \| \| CPV-2c/Dog/Vietnam/HN07/17 \| \| CPV-2c/Pangolin/China/Taiwan/2018/18 \| |
